# Plasma proteome profiling reveals the therapeutic effects of the PPAR pan-agonist chiglitazar on insulin sensitivity, lipid metabolism, and inflammation in type 2 diabetes

**DOI:** 10.1038/s41598-024-51210-8

**Published:** 2024-01-05

**Authors:** Xingyue Wang, You Wang, Junjie Hou, Hongyang Liu, Rong Zeng, Xiangyu Li, Mei Han, Qingrun Li, Linong Ji, Desi Pan, Weiping Jia, Wen Zhong, Tao Xu

**Affiliations:** 1 National Laboratory of Biomacromolecules, CAS Center for Excellence in Biomacromolecules, Institute of Biophysics, Chinese Academy of Sciences, Beijing, China; 2 Sino-Danish College, University of Chinese Academy of Sciences, Beijing, China; 3 CAS Key Laboratory of Systems Biology, CAS Center for Excellence in Molecular Cell Sciences, Shanghai Institute of Biochemistry and Cell Biology, Chinese Academy of Sciences, Shanghai, China; 4 Guangzhou National Laboratory, Guangzhou, China; 5 Department of Endocrinology and Metabolism, Peking University People’s Hospital, Beijing, China; 6 Shenzhen Chipscreen Biosciences Co., Ltd, Shenzhen, China; 7 Department of Endocrinology and Metabolism, Shanghai Jiao Tong University Affiliated Sixth People’s Hospital, Shanghai, China; 8 Shandong First Medical University & Shandong Academy of Medical Sciences, Jinan, China

**Keywords:** Proteome informatics, Endocrine system and metabolic diseases

## Abstract

Chiglitazar is a novel peroxisome proliferator-activated receptor (PPAR) pan-agonist, which passed phase III clinical trials and was newly approved in China for use as an adjunct to diet and exercise in glycemic control in adult patients with Type 2 Diabetes (T2D). To explore the circulating protein signatures associated with the administration of chiglitazar in T2D patients, we conducted a comparative longitudinal study using plasma proteome profiling. Of the 157 T2D patients included in the study, we administered chiglitazar to a specific group, while the controls were given either placebo or sitagliptin. The plasma proteomes were profiled at baseline and 12 and 24 weeks post-treatment using data-independent acquisition mass spectrometry (DIA-MS). Our study indicated that 13 proteins were associated with chiglitazar treatment in T2D patients, including 10 up-regulated proteins (SHBG, TF, APOA2, APOD, GSN, MBL2, CFD, PGLYRP2, A2M, and APOA1) and 3 down-regulated proteins (PRG4, FETUB, and C2) after treatment, which were implicated in the regulation of insulin sensitivity, lipid metabolism, and inflammation response. Our study provides insight into the response of chiglitazar treatment from a proteome perspective and demonstrates the multi-faceted effects of chiglitazar in T2D patients, which will help the clinical application of chiglitazar and further study of its action mechanism.

## Introduction

Type 2 diabetes (T2D) is a chronic metabolic disease characterized by insulin resistance, deficient insulin secretion, and hyperglycemia, which results in long-term complications^1^. In T2D patients, reduced insulin release by impaired pancreatic β-cells and glucose uptake by adipose tissue, muscle, and other tissues, as well as increased glucose production in the liver, lead to elevated and sustained high blood glucose levels^2^. Several antidiabetic agents, such as metformin, sulfonylureas (SUs), meglitinides, alpha-glucosidase inhibitors (AGIs), dipeptidyl peptidase-4 (DPP-4) inhibitors, sodium-glucose co-transporter 2 (SGLT2) inhibitors, and thiazolidinediones (TZDs) are utilized in clinical practice to control glucose levels^3^. Previous studies indicate that these agents differ in therapeutic actions: metformin inhibits hepatic gluconeogenesis; SUs, meglitinides, and DPP-4 inhibitors, such as sitagliptin, increase insulin release directly or indirectly; AGIs slow down the digestion and absorption of carbohydrates; SGLT2 inhibitors boost glucose excretion in urine, and TZDs, such as pioglitazone (PGZ) and rosiglitazone (RGZ), have been utilized as effective PPARγ agonists to improve insulin sensitivity^3–7^. Moreover, advancements in nanomedicine are contributing to improved glycemic control with developments such as the microneedle-based patch with glucose-responsive insulin-laden nanoscale vesicles^8^, and adipocyte-targeted plasmid adiponectin (pADN)-based nanoformulation^9^. Additionally, novel drug delivery systems such as PGZ-loaded nanostructured lipid carriers (NLCs) are being developed to increase solubility and stability^10^. Buccal and nasal formulations are also being explored to improve the efficacy and utilization of peptide-based therapies^11^.

Peroxisome proliferator-activated receptors (PPARs) are ligand-activated transcription factors that belong to the nuclear hormone receptor superfamily. They play an important role in modulating the expression of genes involved in glucose and lipid metabolism, inflammation, and fibrogenesis^12–14^. A total of three PPAR isotypes, namely PPARα, PPARβ/δ, and PPARγ, have been discovered to date^15^. These isotypes exhibit broad and isotype-specific tissue expression patterns, functions, and ligand-binding properties^12,15^. Due to their excellent druggability and metabolic regulation capabilities, PPARs have been developed as therapeutic targets in metabolic diseases, including dyslipidemia and T2D^13^. Although single-PPAR agonists like TZDs are effective, they are associated with several adverse effects such as edema, bone fractures, and heart failure, which largely restricts the extended use for the long-term management of T2D patients^16–18^.

A strategy to improve efficiency and reduce side effects of current PPAR agonists is to develop dual- or pan-PPAR agonists targeting multiple PPARs to achieve a balanced efficacy-tolerability profile for the treatment of T2D patients^19^. Chiglitazar, as a novel PPAR pan-agonist, represents an advancement in the treatment of T2D by activating all three PPAR isotypes, thus improving therapeutic efficiency and minimizing side effects^20^. Previous in vitro studies have shown that chiglitazar can regulate insulin resistance-associated PPARγ phosphorylation, and modulate the upregulation of *ANGPTL4* and *PDK4*, which are part of the PPAR signal pathway and involved in glucose and lipid metabolism^20,21^. Chiglitazar exhibits distinguishable higher efficiency in reducing blood glucose levels in T2D patients in two randomized, double-blind, placebo- or sitagliptin-controlled, phase III trials^22,23^, and has been approved for glycemic control joining exercise and diet control for adult T2D patients in China since 2021.

In this study, we performed data-independent acquisition mass spectrometry (DIA-MS) to evaluate the therapeutic effects and molecular mechanisms triggered by the administration of chiglitazar. We investigated the longitudinal plasma proteomics profiles of 157 T2D patients enrolled in two randomized phase III chiglitazar clinical trials^22,23^. T2D Patients were administered chiglitazar (N = 103), sitagliptin (N = 31), or placebo (N = 23), and the plasma proteome was profiled at baseline, 12 and 24 weeks of treatment, respectively. This investigation aimed to deepen our understanding of the therapeutic effects of chiglitazar, thereby supporting its development for therapeutic strategies in T2D.

## Results

### Efficacy of chigltazar on blood glucose reduction

**Figure 1:**
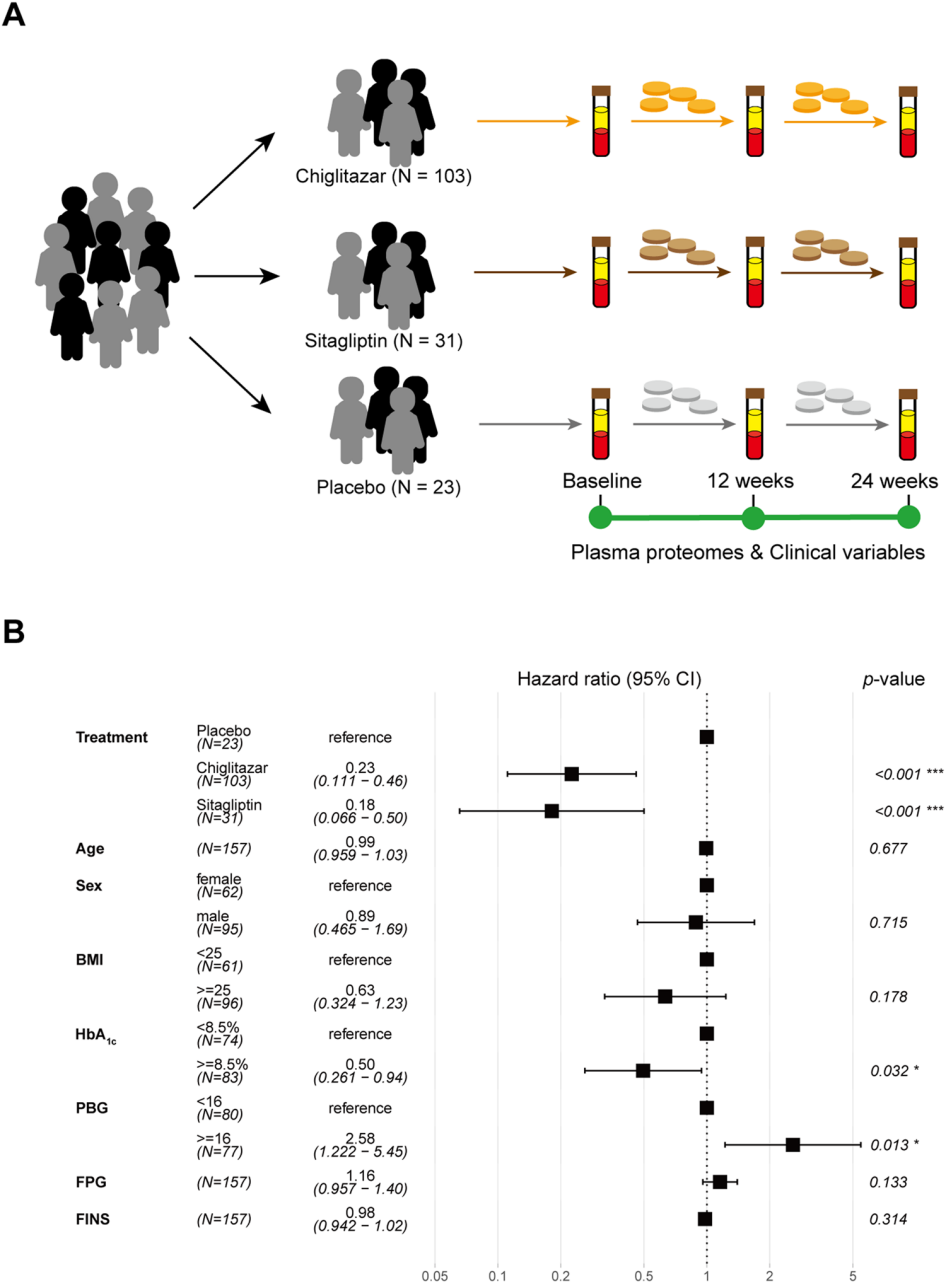
Overview of the study design and the multivariate Cox regression analysis of different parameters on blood glucose-lowering effects. (**A**) Patients who met the filter criteria took chiglitazar (N = 103), sitagliptin (N = 31), or placebo (N = 23) for 24 weeks, and plasma samples and clinical variables were collected and measured at 3 time points, including before the first time taking the drug (baseline) and every 12 weeks after daily medication intake. (**B**) Forest plot showing multivariate Cox regression analysis of the effect of treatment, age, sex, body mass index (BMI), and baseline level of blood glucose parameters on blood glucose-lowering effects (N = 157). PBG, postprandial blood glucose; FPG, fasting plasma glucose; FINS, fasting insulin.

A total of 157 T2D patients with complete blood sampling at baseline and post-treatment (12 and 24 weeks) were selected from the two randomized phase III chiglitazar clinical trials^22,23^. These patients were divided into three groups based on their treatment: chiglitazar (N = 103), sitagliptin (N = 31), and placebo (N = 23) for comparative analysis (Fig. 1A). The mean age of these patients was 52.3 years old (26–70 years old), with an average BMI of 26.17 kg/m^2^ (18.5–33.8), and 60.5% were males. No significant differences in clinical characteristics at baseline were observed among the chiglitazar, sitagliptin, and placebo treatment groups (Tables S1 and S2). Associations of drug treatments, age, sex, BMI, and admission blood glucose level with reduced hemoglobin A1c (HbA_1c_) were assessed using multivariate Cox regression analysis. The results indicated that both chiglitazar and sitagliptin significantly reduced the blood glucose levels in T2D patients when compared to placebo (chiglitazar *p* < 0.001; sitagliptin* p* < 0.001) (Fig. 1B). A high initial HbA_1c_ level (≥ 8.5%) is a significantly beneficial factor for reducing blood glucose level (*p* < 0.05, hazard ratio 0.5, 95% confidence interval 0.26–0.94). In contrast, a high postprandial blood glucose (PBG) level (≥ 16 mmol/L) is a significantly unfavorable factor for lowering blood glucose levels (*p* < 0.05, hazard ratio 2.58, 95% confidence interval 1.22–5.45). No significant associations between other clinical characteristics and the reduced level of blood glucose were observed (Fig. 1B).

### DIA-MS-based plasma proteome profiling

**Figure 2:**
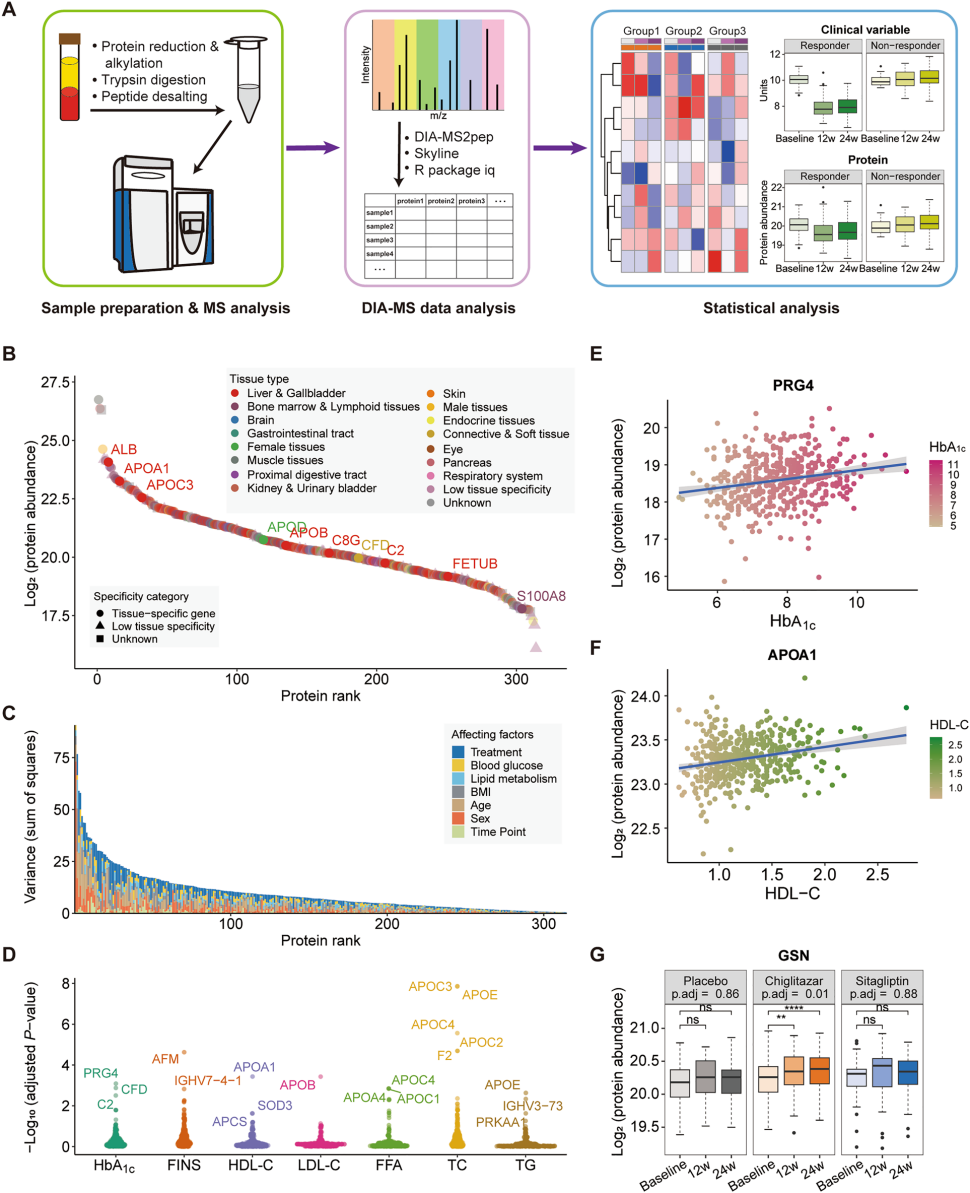
Overview of proteomics profiling upon chiglitazar, sitagliptin, and placebo treatments by data-independent acquisition mass spectrometry. (**A**) Workflow of proteome profiling and data analysis. Plasma sample preparation was performed, and the peptides prepared were injected into the mass spectrometer by applying a data-independent acquisition (DIA) approach. Raw mass spectrometry data were analyzed by applying DIA-MS2pep, Skyline, and the R package iq. After quality control and preprocessing, quantitative proteomics data were used for statistical analysis. (**B**) A total of 314 proteins were quantified from 471 plasma samples and annotated according to HPA (Human Protein Atlas). Tissue-specific categories and enriched tissue types are distinguished. Represent immune-related proteins and tissue-specific proteins are labeled. (**C**) Bar plot showing protein abundance variance explained by treatment, blood glucose, lipid metabolism, and demographics (BMI, age, and sex) measured as the Sum of Squares by using multifactor ANOVA (N = 157). The variance caused by blood glucose (HbA_1c_, PBG, FPG, and FINS) and lipid profiles (HDL-C, LDL-C, FFA, TC, and TG) is shown by the average variance of corresponding clinical variables. (**D**) Results from multifactor ANOVA based on factors of clinical variables, showing the most highly associated proteins with each factor (N = 157). Example of (**E**) an HbA_1c_-associated protein proteoglycan 4 (PRG4), and (**F**) an HDL-C-associated protein apolipoprotein A1 [APOA1; multifactor ANOVA with Benjamini and Hochberg (BH) correction, adjusted *p*-value < 0.05, N = 157]. (**G**) Boxplot showing the protein abundance of the blood glucose- and lipid profile-associated protein gelsolin (GSN) at baseline, week 12, and week 24 in the placebo, chiglitazar, and sitagliptin groups. The asterisks represent the significant degree of change between baseline and week 12 or week 24 (paired *t*-test; placebo, N = 23; chiglitazar, N = 103; sitagliptin, N = 31). *p < 0.05, **p < 0.01, ***p < 0.001, ****p < 0.0001; ns, no significant difference.

The DIA-MS approach was employed for generating plasma proteome profiling with the resulting quantitative proteome data used for subsequent analysis (Fig. 2A, details in methods)^24^. In total, 314 unique proteins that were quantified in all plasma samples passed quality control. Among these proteins, 255 (81.2%) are tissue-specific according to Human Protein Atlas (HPA)^25^, including 119 (37.9%) liver-enriched proteins, 92 (29.3%) lymphoid tissue-enriched proteins, and 3 pancreas-enriched proteins (Fig. 2B and Fig. S1A). Detailed information on all 314 analyzed proteins is provided in Tables S3 and S4. Albumin (ALB) was one of the most abundant proteins as expected (Fig. 2B). Several proteins involved in the PPAR signaling pathway were identified, including apolipoproteins APOA1 and APOA2. In addition, 155 immune-related proteins, such as C8G, C2, CFD, and S100A8, were detected (Fig. 2B).

Multifactor analysis of variance (ANOVA) for the 314 proteins was performed to investigate associations between protein expression and clinical variables, as well as antidiabetic treatments (Fig. 2C,D, and S2; Table S5). The variance in the abundance of 207 proteins was mainly caused by age, sex, and BMI (Fig. 2C). Seven immune and glucose homeostasis-related proteins, including PRG4, CFD, C2, APOA2, GSN, TNXB, and SHBG, were found to have significant associations with HbA_1c_ (Fig. 2D). The association between levels of proteoglycan 4 (PRG4) and HbA_1c_ is visualized in Fig. 2E as an example. Afamin (AFM) was found to be positively associated with fasting insulin (FINS), and it has been reported to be associated with T2D^26^. Five apolipoproteins, including APOA1, APOB, APOC1, APOC3, and APOE, were significantly associated with lipid metabolism (Fig. 2D). APOA1, a major component of high-density lipoprotein (HDL) and involved in reverse cholesterol transport^27^, was positively associated with HDL-cholesterol (HDL-C) (Fig. 2F). Interestingly, we detected five proteins significantly associated with both blood glucose and lipid profiles, including PRG4, APOD, GSN, AZGP1, and LGALS3BP. Three of them (GSN, PRG4, and APOD) were significantly associated with chiglitazar treatment, but not with sitagliptin or placebo treatment. Plasma levels of gelsolin (GSN) in different treatment groups before and after 12 weeks and 24 weeks of treatment are shown in Fig. 2G.

### Plasma proteomics alteration related to chiglitazar treatment

**Figure 3:**
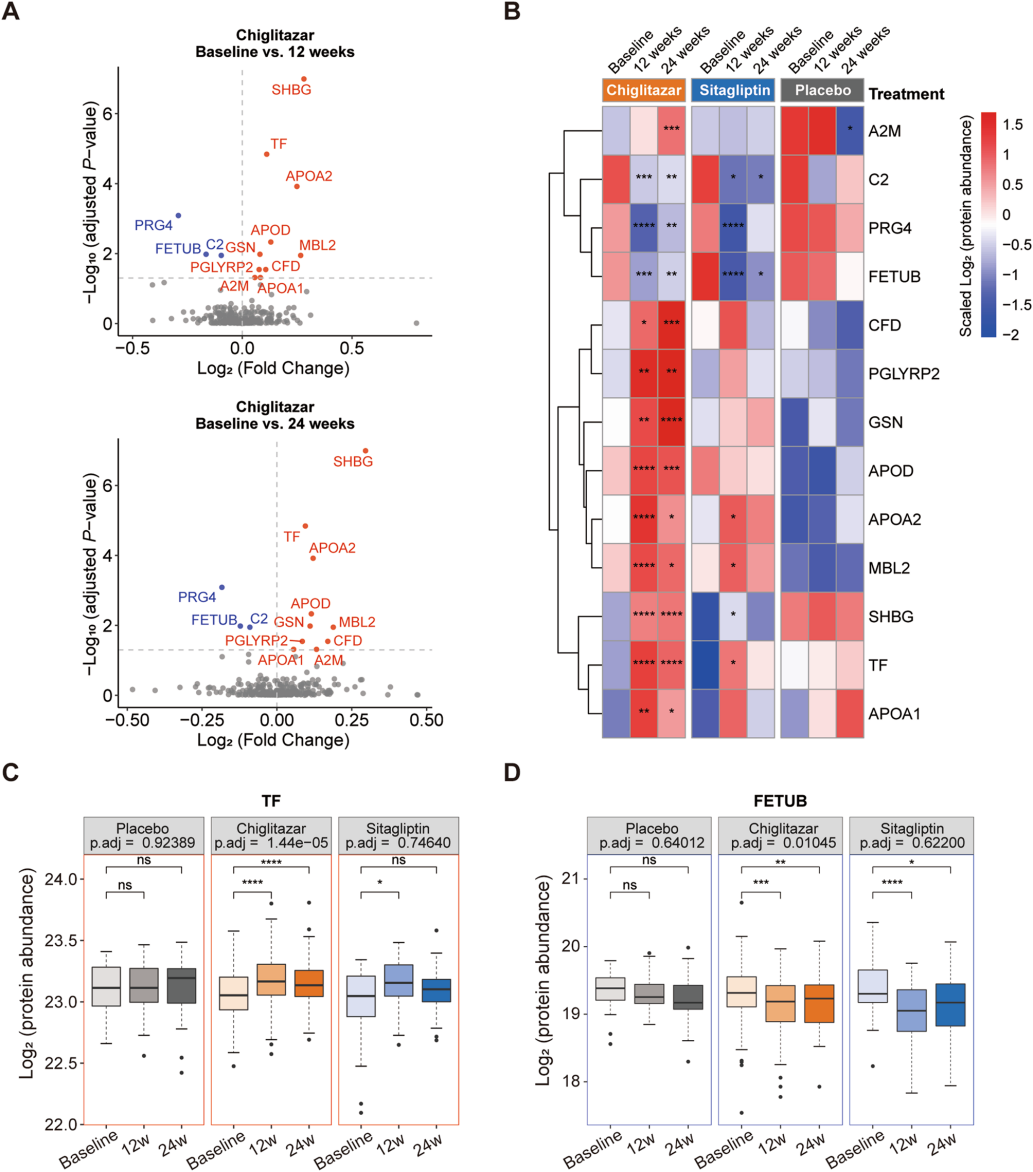
Changes in expression of chiglitazar treatment-associated proteins over treatment time in chiglitazar-, sitagliptin-, and placebo-treated groups. (**A**) Volcano plot with differentially expressed proteins between baseline and post-treatment (12 and 24 weeks) in the chiglitazar group showing the abundance difference on the x-axis and -Log_10_ (adjusted *p*-value) on the y-axis (within-subjects ANOVA with BH correction, N = 103). Significantly upregulated (red) and downregulated (blue) proteins after chiglitazar treatment are shown. (**B**) Heatmap showing plasma levels of 13 chiglitazar treatment-associated proteins (within-subjects ANOVA with BH correction, adjusted *p*-value < 0.05, N = 103) at baseline, week 12, and week 24 in the placebo, chiglitazar, and sitagliptin groups, clustered based on the plasma levels in the 13 proteins. Boxplots showing Log_2_ protein abundance in plasma of (**C**) an upregulated protein transferrin (TF), and (**D**) a downregulated protein fetuin B (FETUB) at baseline, week 12, and week 24 of treatment in the placebo, chiglitazar, and sitagliptin groups. The asterisks represent the significant degree of change between baseline and week 12 or week 24 (paired *t*-test; placebo, N = 23; chiglitazar, N = 103; sitagliptin, N = 31).

To further investigate protein profiles associated with chiglitazar treatment, within-subjects ANOVA with Benjamini and Hochberg (BH) correction was performed for all 314 proteins. In total, 13 proteins were found to be significantly changed after 12- or 24-week treatment of chiglitazar compared to the baseline, including 10 up-regulated proteins (SHBG, TF, APOA2, APOD, GSN, MBL2, CFD, PGLYRP2, A2M, and APOA1) and 3 down-regulated proteins (PRG4, FETUB, and C2) (Fig. 3A and Table S6). Among these proteins, APOA1, APOA2, and APOD are involved in the PPAR signaling pathway and lipid transport, which validates that chiglitazar regulates blood glucose levels by regulating these two pathways.

The heatmap in Fig. 3B illustrates the changes in plasma levels of these 13 proteins at baseline and after 12 and 24 weeks of chiglitazar treatment, alongside comparisons with the sitagliptin and placebo groups. No or less significant changes were identified in the sitagliptin and placebo groups through within-subjects ANOVA for the 13 proteins. Sex hormone-binding globulin (SHBG), which has been reported as a favorable marker for indicating insulin sensitivity^28–32^, was significantly upregulated by the chiglitazar treatment, but less significantly upregulated by the treatment of sitagliptin (Fig. 3A,B, and S3A). Several proteins involved in the PPAR signaling pathway and lipid transport, including APOA1, APOA2, and APOD, were increased after chiglitazar treatment. Additionally, inflammation-related proteins, such as C2 and PGLYRP2, were significantly regulated in the chiglitazar group, whereas the overall trends of these proteins were less significant in the sitagliptin and placebo groups (Fig. 3A,B, and S3A). In addition, we observed significant regulation of histidine-rich glycoprotein (HRG) in the chiglitazar group using a paired *t-*test. Fig. S3B depicts a significant upward trend of HRG in the chiglitazar group, with a less significant downward trend observed in the sitagliptin group.

Transferrin (TF) plays an important part in iron transport and cellular iron uptake^33^, and TF levels are normally lower in T2D patients, which is associated with end-stage renal disease (ESRD)^34,35^. TF levels were significantly increased in T2D patients with either 12- or 24- weeks of chiglitazar treatment while only a slight increase in T2D patients with 12- weeks of sitagliptin treatment (Fig. 3C). Moreover, fetuin B (FETUB), which has been reported as an unfavorable marker for indicating insulin sensitivity^36,37^, was significantly down-regulated by the treatment of both chiglitazar and sitagliptin at week 12 or 24 compared to baseline (Fig. 3D).

### Plasma proteome profiling reveals multi-faceted effects of chiglitazar

**Figure 4:**
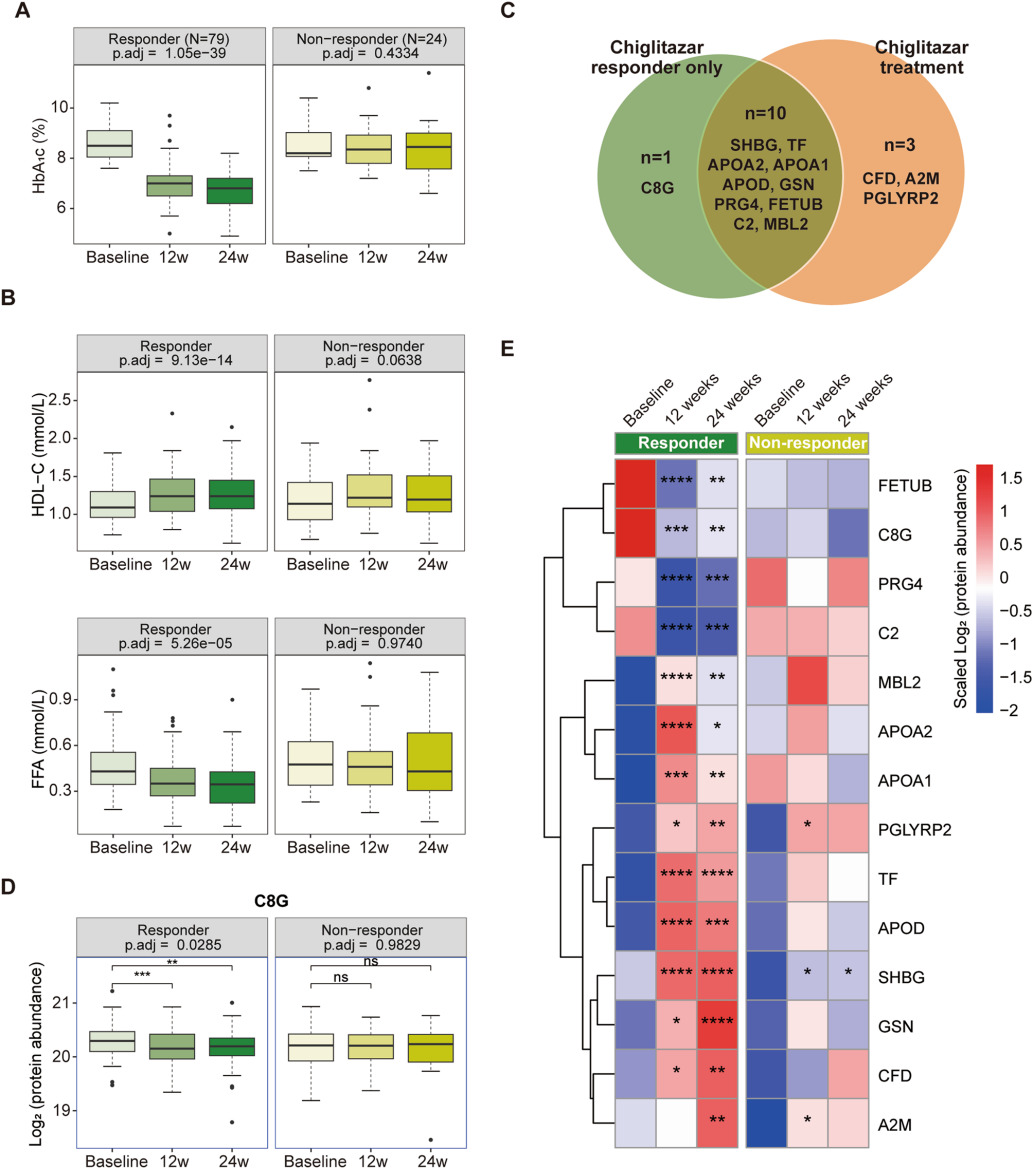
The changes of blood parameters and differentially expressed proteins associated with chiglitazar treatment in the responders. Boxplot showing the changes of (**A**) HbA_1c_, (**B**) high-density lipoprotein cholesterol (HDL-C) and free fatty acids (FFA) over treatment time in the chiglitazar responder and non-responder groups [within-subjects ANOVA with Benjamini and Hochberg (BH) correction, adjusted *p*-value < 0.05; responder, N = 79; non-responder, N = 24]. (**C**) Venn plot showing the overlap of significant expression changed proteins before and after removing chiglitazar non-responders in chiglitazar treated T2D patients. (**D**) Boxplots showing Log_2_ protein abundance in plasma of complement C8 gamma chain (C8G) at baseline, week 12 and week 24 of treatment in the responder and non-responder groups. The significant degree of change is labeled (within-subjects ANOVA with BH correction; responder, N = 79; non-responder, N = 24). (**E**) Heatmap showing the chiglitazar-regulated protein levels at baseline, week 12, and week 24 of treatment in the chiglitazar responder and non-responder groups, clustered based on the plasma levels. The asterisks represent the significant degree of change between baseline and week 12 or week 24 (paired *t*-test; responder, N = 79; non-responder, N = 24).

To further explore chiglitazar treatment response, clinical variable changes, and plasma proteome alternations in T2D patients, we divided the patients into chiglitazar responder (N = 79) and non-responder (N = 24) groups based on the decreased levels of HbA_1c_ after 24 weeks of treatment (Fig. 4A and Methods). A within-subjects ANOVA demonstrated that certain lipid profiles, such as HDL-C, free fatty acids (FFA) and triglycerides (TG), and glucose variable FINS, exhibited significant changes exclusively in the responder group (Fig. 4B and Table S7). Elevated lipid profiles total cholesterol (TC) and LDL-C and declined glucose variables fasting plasma glucose (FPG) and PBG showed more significant changing trends in the responder groups (Table S7). The changing trends of these clinical variables observed in the responder group are consistent with the phase III clinical trials of chiglitazar^22,23^.

The different clinical variable changes between chiglitazar responders and non-responders shed light on the differential drug response between individuals, bringing the question of whether the differences exist at the proteome level. Thus, proteins with significant associations with chiglitazar treatment response were also analyzed using within-subjects ANOVA and 11 differentially expressed proteins were identified (Fig. S4 and Table S8). The shared or unique differentially expressed proteins between the chiglitazar responders and chiglitazar-treated T2D patients were analyzed. We found that 10 proteins were shared in the two datasets, 1 protein was uniquely included in chiglitazar responders and 3 proteins were uniquely included in chiglitazar-treated T2D patients (Fig. 4C). Interestingly, we found that the inflammation-related protein, C8 gamma chain (C8G), exhibited a notable downward trend only in chiglitazar responders but not the non-responders (Fig. 4D). The three chiglitazar treatment-associated proteins CFD, PGLYRP2, and A2M, which were not identified when compare the responders and the non-responders using within-subjects ANOVA, also showed more obvious expression changes after treatment in the responder groups by paired *t*-test (Fig. 4E).

The expression changes of these proteins helped explain the blood glucose and lipid-related clinical variable changes and revealed the multi-faceted effects of chiglitazar. Six proteins function in regulating glucose homeostasis, insulin resistance, and β-cell function, including SHBG, FETUB, PRG4, GSN, CFD, and TF, among which SHBG and FETUB are markers of insulin sensitivity^37–40^ and the expression of *TF* in adipose tissue is positively associated with insulin sensitivity^41^. Notably, TF and GSN also play roles in regulating kidney functions^42,43^, implying the potential renoprotective effect of chiglitazar for T2D patients. Three apolipoproteins APOA1, APOA2, and APOD, which were significantly up-regulated after chiglitazar treatment, might contribute to lipid profile changes via regulating lipid transport and metabolism^27,44^. Besides, chiglitazar has a potential inflammation regulation effect with 6 chiglitazar treatment-related proteins, including C2, C8G, CFD, MBL2, A2M, and PGLYRP2, largely enriched in the complement and coagulation cascade pathways and regulate inflammation^45–47^. Altogether, the consistency of changes in clinical variables and proteins in chiglitazar responders supports the glycemic and lipid control effects of chiglitazar reported in the phase III clinical trials and further reveals the potential inflammation regulation and kidney protection effects at the proteome level.

## Discussion

T2D is a complex chronic metabolic disorder, resulting from an interplay of multiple genetic and environmental factors. In recent years, the management of T2D patients has undergone a major conceptual change, with treatment objectives shifting from primarily focusing on glucose management to adopting a more patient-centered approach, considering patients’ physiological and pathological conditions, in selecting appropriate pharmacologic treatment^48^. Chiglitazar is a novel PPAR pan-agonist and has shown benefits in both hypoglycemic control and lipid regulation in T2D patients as evidenced by previous clinical trials^22,23^. The advantage of a pan-PPAR agonist is its ability to target all three PPAR isoforms (α, γ, and δ), which may enable a more comprehensive and balanced therapeutic effect on glucose and lipid metabolism^22,23^, while also influencing inflammation and fibrogenesis^49^. Nevertheless, further investigation of the therapeutic effects of chiglitazar is needed to understand the potential molecular impacts and the corresponding mechanisms.

Our results revealed the multi-faceted effects of chiglitazar in T2D treatment using a systematic analysis of plasma proteome profiling in T2D patients, and the correlation between clinical variables and proteins exclusively observed in chiglitazar responder groups further provides support for the glycemic control and lipid regulation effect of chiglitazar reported in clinical trials.

We found that the proteins involved in glucose homeostasis, insulin resistance, and β-cell function changed significantly. One of the most significantly changed proteins, SHBG, has been found to be strongly associated with glucose homeostasis^50^. Downregulation of SHBG is a strong predictor of T2D^38,40^, and up-regulation was observed after chiglitazar treatment. A hepatokine, FETUB, which is regulated by steatosis and causes glucose intolerance by modulating insulin-independent glucose metabolism and is upregulated in T2D patients^37,39^, was also significantly downregulated after chiglitazar treatment. The proteins CFD, GSN, and PRG4, which play important roles in regulating glucose homeostasis and β-cell function^51–54^, were significantly changed after chiglitazar treatment. These findings suggest that chiglitazar improves the key proteins involved in blood glucose regulation in T2D patients, proving the glycemic control effect from the view of proteomics.

Chiglitazar modulates lipid transport and hepatic lipid metabolism in T2D patients. Three apolipoproteins, APOA1, APOA2, and APOD, were significantly upregulated after chiglitazar treatment. APOA1 and APOA2, which are the major protein components of HDL and encoded by target genes of PPARα, promote reverse cholesterol transport from peripheral tissues to the liver^27,55^. APOD binds cholesterol and removes redundant cholesterol by reverse transport via HDL^44,56^. In addition to these apolipoproteins, other significantly changed proteins suggest a beneficial effect of chiglitazar on hepatic lipid metabolism. It has been reported that the hepatokine SHBG is associated with the stimulatory regulation of hepatocyte nuclear factor 4 (HNF-4) and inhibitory regulation of PPARγ^57,58^. The observed upregulation of SHBG might result from the inhibition of PPARγ, providing evidence for the effectiveness of chiglitazar as a hypoglycemic agent and its ability to decrease de novo lipogenesis in the liver. Furthermore, the glucose intolerance-related protein PRG4, which affects hepatic steatosis and was found to be down-regulated after bariatric surgery^51,59^, showed the same significant change in our study. These findings support the lipid-modulating effect of chiglitazar over sitagliptin from a proteome perspective and indicate the underlying regulatory mechanism and the potential for the treatment of non-alcoholic fatty liver disease (NAFLD) with related clinical trials ongoing.

Except for glucose and lipid dysregulation, chronic inflammation is also associated with T2D, contributing to macrovascular and microvascular complications^60^. Inflammation-related proteins were significantly altered by chiglitazar treatment, indicating its inflammatory regulation effect. Five chiglitazar treatment-associated proteins, including CFD, C8G, A2M, C2, and MBL2, are enriched in the complement and coagulation cascade pathways. Downregulation of C2 and C8G suggests a reduction in inflammatory responses in T2D patients^45,46^. Upregulation of peptidoglycan recognition protein 2 (PGLYRP2), a protein preventing overactivation of the immune system and excessive inflammation^47^, was also observed, which is consistent with previous research in T2D and T1D patients^59,61^. Together, these findings provide support for the immunomodulatory effects of chiglitazar in T2D patients.

Chiglitazar has potential renoprotective effects in T2D patients by regulating the expression of related proteins. Studies have shown that low serum TF concentration is associated with ESRD in T2D patients ^35^. Although the mechanism is not fully understood, elevated TF levels suggest a protective effect on the kidneys. Decreased GSN may lead to kidney injury by promoting activation of protein kinase C (PKC)^42,62^, and the elevation in GSN observed after chiglitazar treatment also suggests a renoprotective effect. Additionally, the protein HRG, which may have a protective effect against diabetic tubulointerstitial injury^63^, was up-regulated in the chiglitazar group but changed oppositely in the sitagliptin group. This suggests that compared to sitagliptin, chiglitazar provides additional renal protection benefits beyond its lipid-modulating effects.

Sitagliptin was the only oral antidiabetic agent that had undergone randomized controlled clinical trials in China when the study was initially designed^64^. Given the potential therapeutic applications of chiglitazar and sitagliptin are similar, with metformin as the first-line drug^22^, sitagliptin was deemed an appropriate active comparator in the study. Different plasma proteome changes in T2D patients under the treatment of chiglitazar or sitagliptin reveal the similarities and differences in their effects and mechanisms. PRG4 and FETUB, both playing roles in regulating insulin sensitivity^37,39,51^, were significantly downregulated after 12 weeks of chiglitazar or sitagliptin treatment, which suggests the common mechanism of lowering blood glucose levels. The differences in plasma proteome changes highlight the advantages of chiglitazar over sitagliptin in regulating lipid metabolism and inflammation, as well as renoprotective effects in T2D patients, since associated proteins, such as APOA1, PGLYRP2, and GSN, were found significantly regulated only after chiglitazar treatment but not sitagliptin treatment. These findings help the personalized medicine selection according to the symptoms and treatment needs of T2D patients.

In this study, we performed a longitudinal plasma proteome analysis in T2D patients who received the treatment of the novel PPAR pan-agonist chiglitazar with placebo and sitagliptin as control groups. Our findings revealed significant changes in proteins related to glucose homeostasis and lipid metabolism. This suggests that chiglitazar effectively contributes to glycemic control and lipid management in T2D patients at the proteome level. Additionally, we observed significant regulatory effects on proteins involved in inflammation and renal protection, thereby highlighting the multi-faceted benefits of chiglitazar. In conclusion, this research gives a comprehensive overview of the therapeutic effects of chiglitazar with revealed protein alternation support clinical findings, which may serve as a foundation for further research on chiglitazar’s working mechanisms and make strides in the clinical use of chiglitazar in T2D patients.

## Methods

### Trial design and participants

The study was designed as extended research of the randomized, double-blind, placebo- or sitagliptin-controlled, phase III trials (CMAP and CMAS) that were registered with ClinicalTrials.gov (NCT02121717 and NCT02173457). Details on the recruitment of study participants and trial design have been described previously^22,23^. In summary, eligible patients were those with T2D who had insufficient glycemic control (HbA_1c_ ≥ 7.5% and ≤ 10.0%) despite adhering to a strict diet and exercise regimen. After 2 weeks of placebo run-in, 1275 patients were randomized to receive placebo, chiglitazar, or sitagliptin. Participants orally took the drug 30 min after breakfast each day. Blood samples and clinical variables were collected before the first drug administration and every 12 weeks thereafter. Among the participants who finished the 24-week double-blind treatment, 103 participants from the chiglitazar, 31 from the sitagliptin group, and 23 from the placebo group were selected for the study.

Ethical approvals were obtained from the ethical committees of Shanghai Sixth People’s Hospital and Peking University People’s Hospital. The study protocol conforms to the guidelines of Good Clinical Practice and the Declaration of Helsinki. All participants provided written informed consent.

### Plasma sample preparation and DIA-MS analysis

A total of 471 plasma samples collected from the 157 patients (chiglitazar, N = 103; sitagliptin, N = 31; placebo, N = 23) at three time points were prepared and analyzed by applying a DIA approach. Ten microliters of each plasma sample were diluted with 8 M urea followed by disulfide reduction and alkylation. Proteins were digested with trypsin overnight at 37 ℃, and the digested peptides were purified and desalted. The desalted peptides were dried under a vacuum and dissolved in MS buffer.

The peptides were separated with a chromatographic duration of 60 min and injected into a Thermo Scientific Q Exactive HF-X Hybrid Quadrupole-Orbitrap (QE HF-X) mass spectrometer. The DIA acquisition scheme consisted of 46 windows ranging from 400 to 900 m*/z*. The window width was 12 Da, with a 0.5 Da margin width. The resolution of MS1 and MS2 was 30,000. The automatic gain control (AGC) of MS1 was 3 × 10^6^; that of MS2 was 1 × 10^5^. The maximum ion injection time (IT) was 50 ms. The raw mass spectrometry data have been deposited to the ProteomeXchange Consortium via the PRIDE partner repository with the dataset identifier PXD039827^65,66^.

The raw files were converted to .mzML format, and database searching was performed by applying the DIA-MS2pep framework for peptide identification^24^. A BLIB library of DIA data was created based on identified peptides, peptide quantification was performed by using Skyline 19.1^67^, and the results were exported for further quantification. The iq R package was used for protein quantification and normalization adopting the top 3 approach^68^, which aggregates the abundance of the top 3 most intense peptides from one protein as the protein abundance. After MS data analysis, 384 proteins were identified from 471 samples.

### Quality control and preprocessing

Proteins with more than 75% missing values among all the samples and not predicted to be secreted into blood based on the human secretome were deleted^69^. Proteins with more than 75% missing values among samples collected at baseline were further deleted because the baseline expression levels of proteins were used as the reference of expression changes of proteins. As a result, 314 of the identified 384 proteins were used for further analysis. Protein measures were log_2_-transformed.

We used the function removeBatchEffect from the R package limma to remove the batch effects caused by the different experimental sites and MS batches^70^, taking into account age, sex, BMI, medication, and treatment time. The average abundances of proteins were not much changed after batch effect removal, and the batch effects of experimental sites and MS were removed effectively (Figs. S1B-E).

### Classification of T2D patients based on drug response

The patients were classified as responders if their lowered HbA_1c_ value at both week 12 and week 24 was no less than 0.5%. Considering that some patients may have delayed responses, the patients were also classified as responders if their lowered HbA_1c_ value was no less than 0.2% at week 12 and no less than 1.5% at week 24. Patients who didn’t meet any of the above two criteria were classified as non-responders. The criteria were based on one secondary efficacy endpoint of the phase III trials, which is the proportion of patients having a lowered HbA_1c_ value of no less than 0.5% at week 24^22,23^.

### Statistical analysis

Multivariate Cox regression analysis for associations of multiple factors with blood glucose-lowering effects was performed using the coxph() function of the survival R package^71^. Multifactor analysis of variance (ANOVA) was performed for association analysis between protein expression and clinical variables as well as treatment using the built-in R function anova(), and variance of protein abundance was measured as the Sum of Squares. Within-subjects ANOVA was performed to detect differentially expressed proteins over treatment time in each group using the anova_test() function of the rstatix R package^72^. Paired *t*-test was used to analyze the difference in protein levels between two time points within a group. Benjamini and Hochberg (BH) correction was used to adjust the *p*-values and the values less than 0.05 were considered statistically significant. All statistical analyses and visualization were performed with R version 4.1.0^73^.

### Supplementary Information

Supplementary Information 1.

Supplementary Information 2.

## Data Availability

The data that support the findings of this study have been deposited to the ProteomeXchange Consortium with the dataset identifier PXD039827.
