# Supplementary material for: Plasma proteome profiling reveals the therapeutic effects of the PPAR pan-agonist chiglitazar on insulin sensitivity, lipid metabolism, and inflammation in type 2 diabetes
Source: Sci Rep. 2024 Jan 5;14:638. doi: 10.1038/s41598-024-51210-8 (PMC10770401; doi:10.1038/s41598-024-51210-8)
Supplement: Supplementary file 2 — Supplementary Information 2. [file 41598_2024_51210_MOESM2_ESM.docx]

**Supplementary figures for**

**Plasma proteome profiling reveals the therapeutic effects of the PPAR pan-agonist chiglitazar on insulin sensitivity, lipid metabolism, and inflammation in type 2 diabetes**

Xingyue Wang^1,2,+^, You Wang^1,+^, Junjie Hou^1,+^, Hongyang Liu^1^, Rong Zeng^3^, Xiangyu Li^4^, Mei Han^4^, Qingrun Li^3^, Linong Ji^5^, Desi Pan^6^, Weiping Jia^7^, Wen Zhong^4*^, Tao Xu^1,4,8,*^

^1^National Laboratory of Biomacromolecules, CAS Center for Excellence in Biomacromolecules, Institute of Biophysics, Chinese Academy of Sciences, Beijing, China

^2^Sino-Danish College, University of Chinese Academy of Sciences, Beijing, China

^3^CAS Key Laboratory of Systems Biology, CAS Center for Excellence in Molecular Cell Sciences, Shanghai Institute of Biochemistry and Cell Biology, Chinese Academy of Sciences, Shanghai, China

^4^Guangzhou National Laboratory, Guangzhou, China

^5^Department of Endocrinology and Metabolism, Peking University People’s Hospital, Beijing, China

^6^Shenzhen Chipscreen Biosciences Co., Ltd., Shenzhen, China

^7^Department of Endocrinology and Metabolism, Shanghai Jiao Tong University Affiliated Sixth People’s Hospital, Shanghai, China

^8^Shandong First Medical University & Shandong Academy of Medical Sciences, Jinan, China

^+^These authors contributed equally: Xingyue Wang, You Wang, Junjie Hou

*Corresponding authors: Wen Zhong, [zhong_wen@gzlab.ac.cn](mailto:zhong_wen@gzlab.ac.cn); Tao Xu, [xutao@ibp.ac.cn](mailto:xutao@ibp.ac.cn)


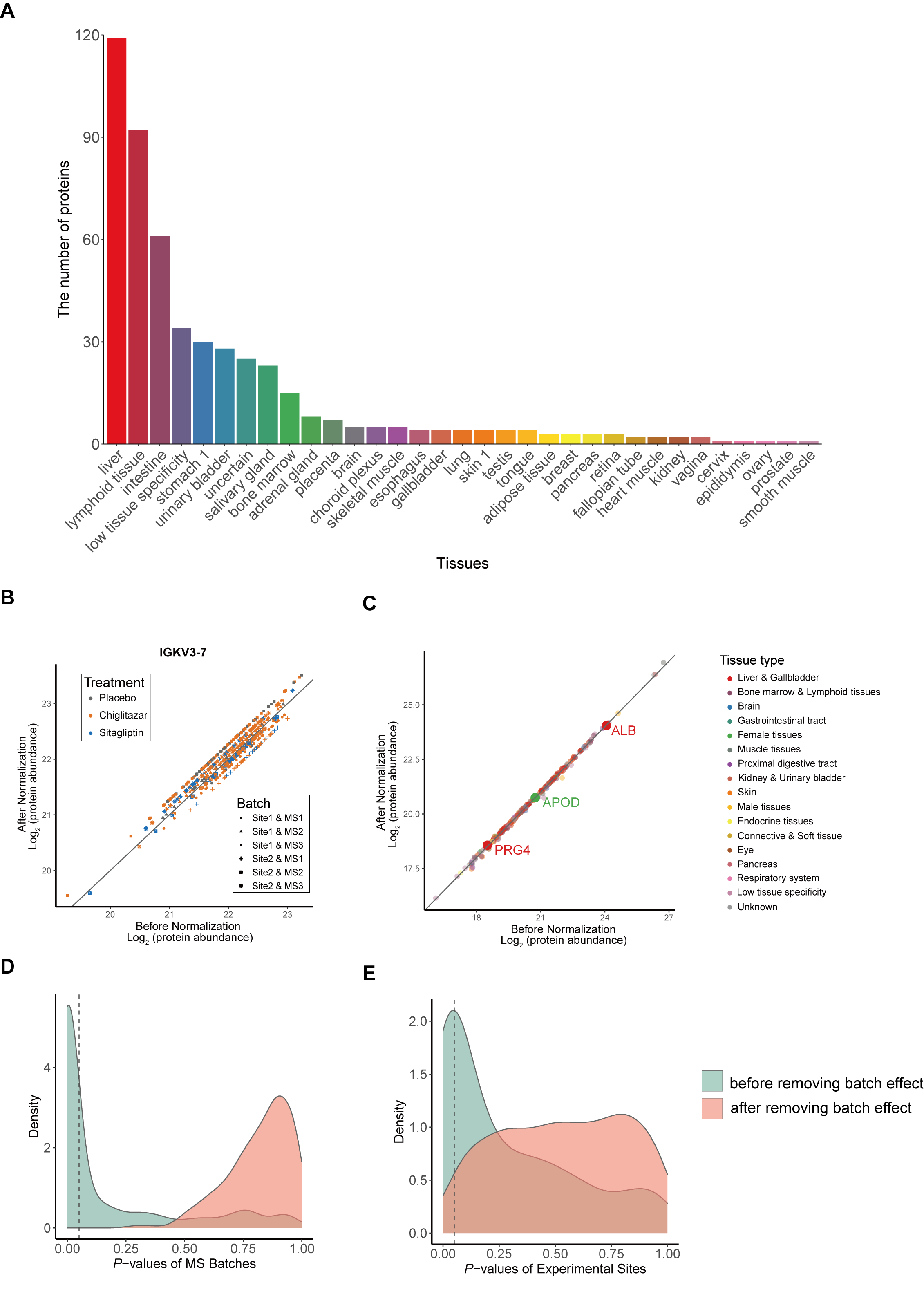


**Figure S1**. Tissue-specific annotation based on Human Protein Atlas (HPA) and data preprocessing.

(**A**) Histogram showing enriched tissues for the 314 identified proteins from 471 plasma samples, as annotated based on Human Protein Atlas (HPA) and ordered by the number of enriched proteins.

(**B**) Scatter plot of an example protein, immunoglobulin kappa variable 3-7 (IGKV3-7), showing protein abundance before and after removing batch effects (normalization) in 471 plasma samples. Samples belonging to different batches (shape of the point) and treatment groups (color of the point) are distinguished.

(**C**) Scatter plot showing changes in average protein abundance in 471 plasma samples before and after removing batch effects. Enriched tissue types of each protein are distinguished by colors, and 3 proteins as examples of the high, medium, and low abundance proteins are labeled.

(**D**) Density plot of *p*-values of mass spectrometry (MS) batches before and after removing batch effects (multifactor ANOVA, N=157).

(**E**) Density plot of *p*-values of experimental site batches (clinical trials) before and after removing batch effects (multifactor ANOVA, N=157).


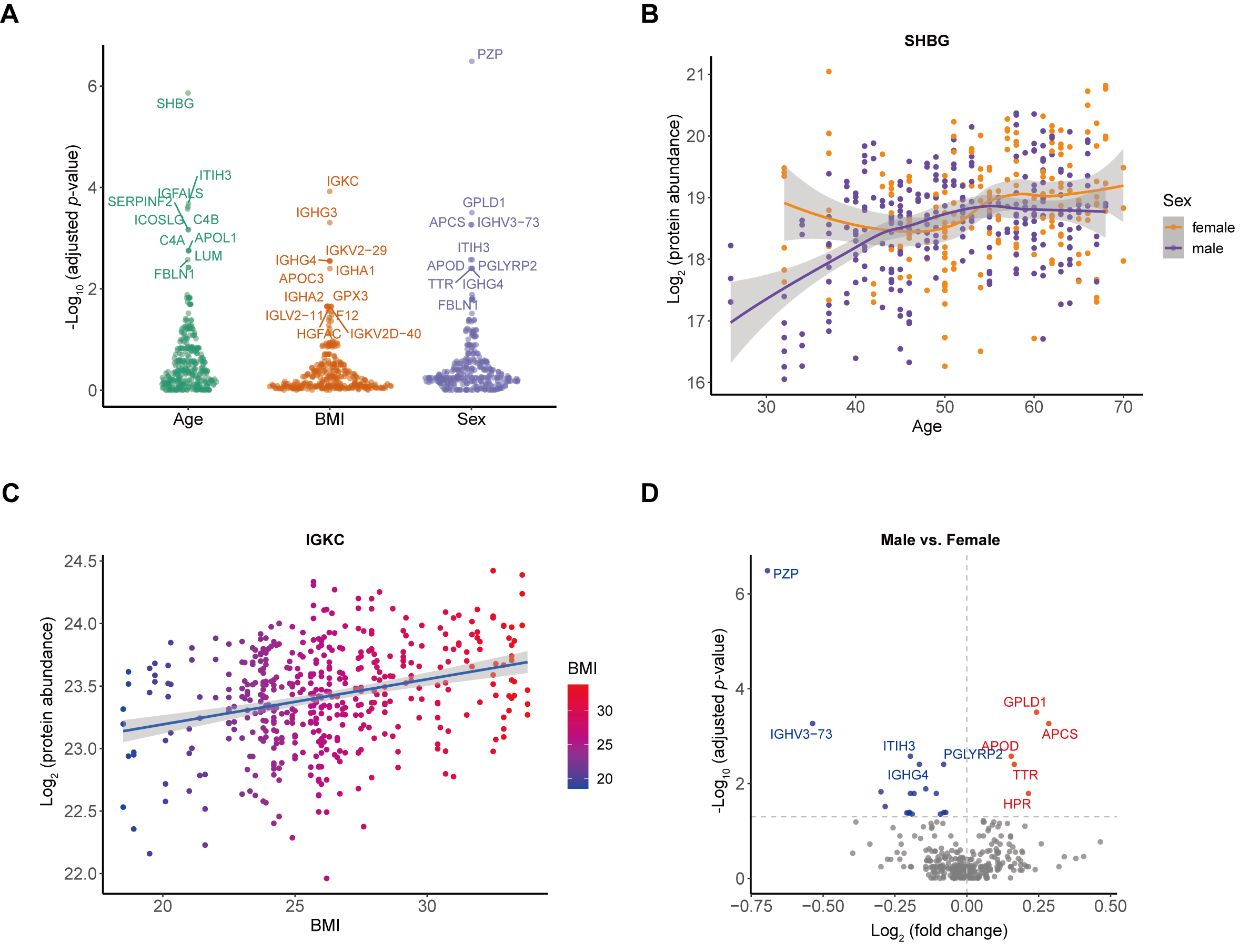


**Figure S2**. Proteins associated with age, body mass index (BMI), and sex.

(**A**) Results from multifactor ANOVA based on the factors age, BMI, and sex, showing the most highly associated proteins with each factor (N=157).

Example of (**B**) an age- and sex-associated protein sex hormone-binding globulin (SHBG), and (**C**) a BMI-associated protein immunoglobulin kappa constant (IGKC) (multifactor ANOVA with BH correction, adjusted *p*-value < 0.05, N=157).

(**D**) Volcano plot with differentially expressed proteins between males and females showing the abundance difference on the x-axis and -Log_10_ (adjusted *p*-value) on the y-axis (multifactor ANOVA with BH correction, N=157). Proteins significantly upregulated in males (red) or females (blue) are shown.


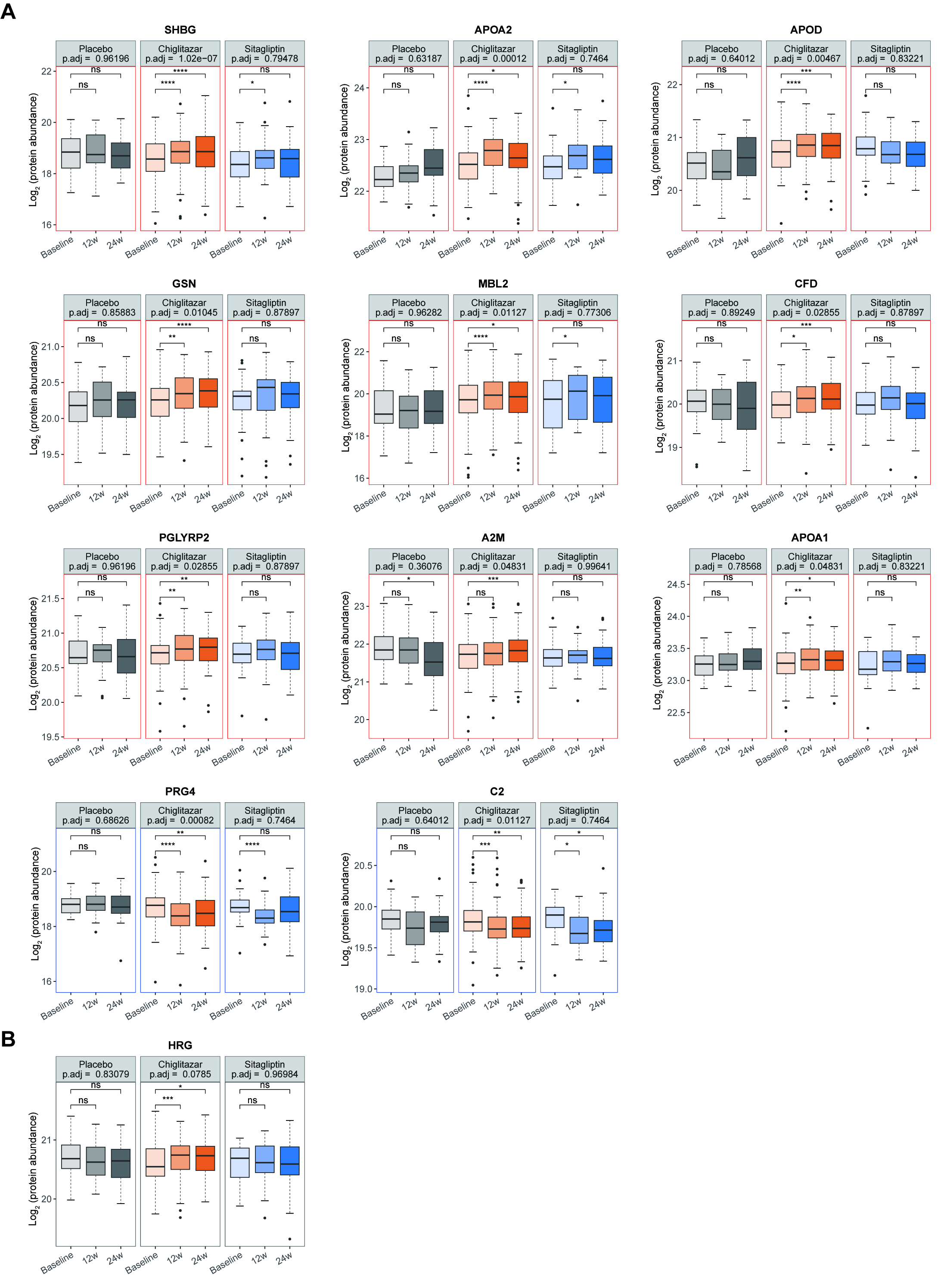


**Figure S3**. Changing trends and significant levels of proteins in different treatment groups.

(**A**) Boxplots showing plasma levels of nine upregulated proteins (red frame) and two downregulated proteins (blue frame) after chiglitazar treatment (within-subjects ANOVA with BH correction, adjusted *p*-value < 0.05, N=103) at baseline, week 12, and week 24 in the placebo, chiglitazar, and sitagliptin groups.

(**B**) Boxplot showing plasma levels of histidine-rich glycoprotein (HRG) at baseline, week 12 and week 24 in the placebo, chiglitazar, and sitagliptin groups with adjusted *p*-values labeled (within-subjects ANOVA with BH correction; placebo, N=23; chiglitazar, N=103; sitagliptin, N=31). The asterisks represent the significant degree of change between baseline and week 12 or week 24 (paired *t*-test; placebo, N=23; chiglitazar, N=103; sitagliptin, N=31). **p*<0.05, ***p*<0.01, ****p*<0.001, *****p*<0.0001; ns, no significant difference


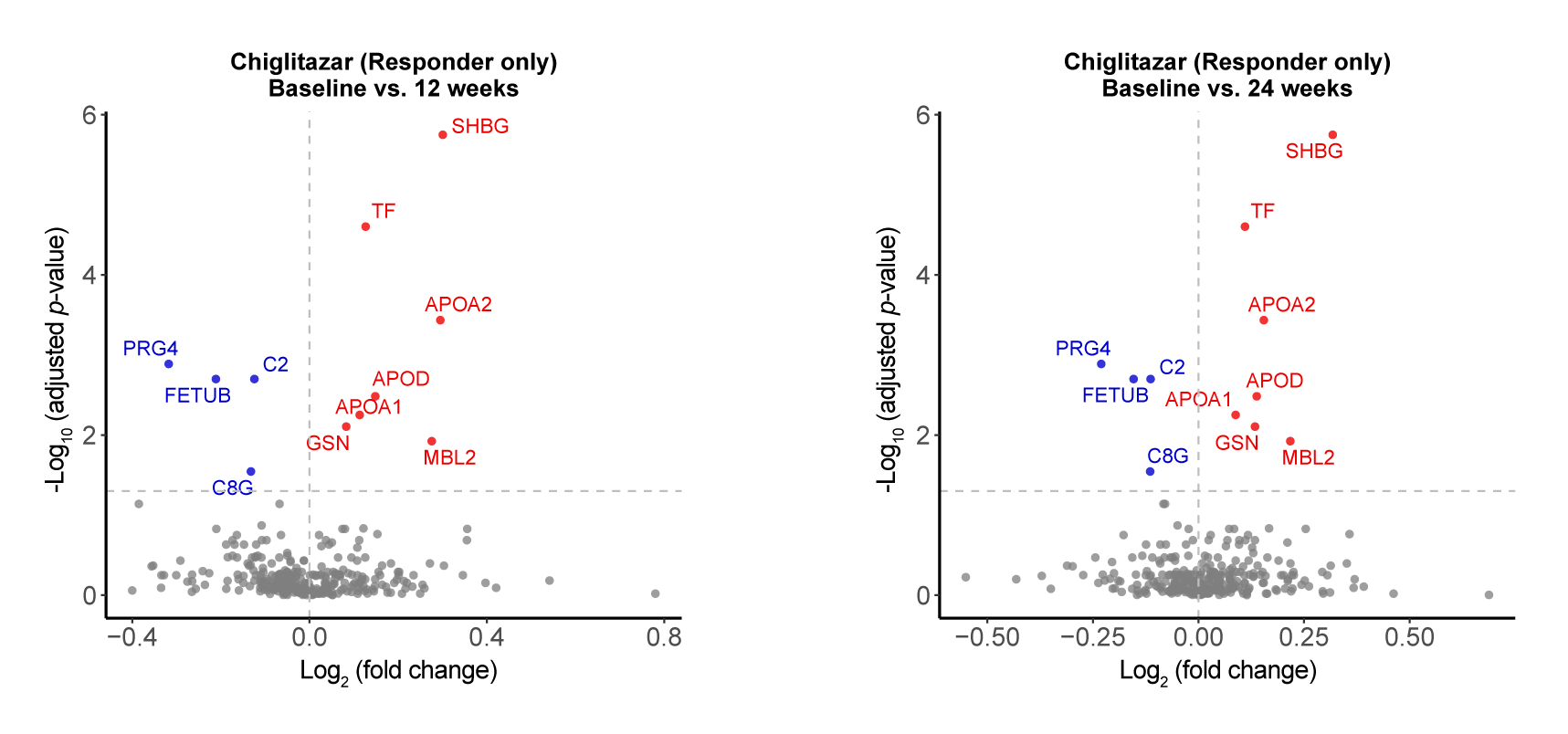


**Figure S4**. Expression changes of chiglitazar treatment response-associated proteins

Volcano plot with differentially expressed proteins between baseline and posttreatment (12 and 24 weeks) in the chiglitazar responder group showing the abundance difference on the x-axis and -Log_10_ (adjusted *p*-value) on the y-axis (within-subjects ANOVA with BH correction, N=103). Significantly upregulated (red) and downregulated (blue) proteins after chiglitazar treatment are shown.
